# Characterization of E-Cadherin, SSEA-1, MSI-1, and SOX-2 Expression and Their Association with Pale Cells in Adenomyosis

**DOI:** 10.3390/biom14111355

**Published:** 2024-10-24

**Authors:** Jingjun Tian, Veronika Hoffmann, Mohamed Gamal Ibrahim, Uwe Hansen, Andreas N. Schüring, Renata Voltolini Velho, Sylvia Mechsner, Martin Götte

**Affiliations:** 1 Department of Gynecology Charité with Center of Oncological Surgery, Endometriosis Research Center Charité, Campus Virchow-Klinikum, Augustenburger Platz 1, 13353 Berlin, Germany; jingjun.tian@charite.de (J.T.); mgs_medicine@hotmail.com (M.G.I.); renata.voltolini-velho@charite.de (R.V.V.); 2 Department of Gynecology and Obstetrics, Münster University Hospital, Labor PAN-Zentrum, Vesaliusweg 2–4, 48149 Münster, Germany; v.altenburger@gmx.de (V.H.); aschuering@kitz-regensburg.de (A.N.S.); 3 Team Kinderwunsch Oldenburg, 26121 Oldenburg, Germany; 4 Institute of Musculoskeletal Medicine, University Hospital Münster, 48149 Münster, Germany; uhansen@uni-muenster.de; 5 Fertility Centre KITZ Regensburg, 93047 Regensburg, Germany; 6 Cells in Motion Interfaculty Centre, University of Münster, 48149 Münster, Germany

**Keywords:** adenomyosis, pale cell, E-cadherin, SSEA-1, SOX-2, MSI-1

## Abstract

Adenomyosis (AM) is a gynecological disease characterized by the invasion of endometrial glands and stroma within the myometrium. The etiology and pathogenesis of AM remain inadequately understood. Pale cells were identified as a novel cell type characterized by the absence of desmosomal contacts and light-colored cytoplasm. These cells were observed to migrate individually through ultra-micro ruptures in the basal membrane of the endometrial glands, translocating into the stroma and then further into the myometrium. Our study aimed to explore the possible stem cell properties of these pale cells. Forty hysterectomy specimens were analyzed using immunohistochemistry and immunofluorescence to assess negative E-cadherin expression and the positive expression of stem cell markers SSEA-1, MSI-1, and SOX-2. Immunohistochemical analysis revealed the presence of pale cells and occasionally rounded, enlarged E-cadherin-negative cells predominantly in the basal endometrial epithelium. The stem cell marker SSEA-1 was significantly elevated in the basalis epithelium, as well as in the ectopic epithelium. SSEA-1 positive cells were also identified in the stroma and myometrium. Sporadic colocalization of SSEA-1+/E-cadherin– cells was confirmed through immunofluorescence. The positive staining of pale cells for SSEA-1 and MSI-1 was also confirmed at the ultrastructural level by immunoelectron microscopy. These findings indicate that pale cells may possess stem cell characteristics, particularly a positive SSEA-1 profile, warranting further in vitro investigation into their role in the pathogenesis of adenomyosis.

## 1. Introduction

Adenomyosis (AM) describes the benign invasion of endometrium-like tissue into the myometrium, resulting in a diffusely enlarged uterus with microscopic ectopic non-neoplastic endometrial glands and stroma surrounded by hypertrophic–hyperplastic musculature [1,2]. The associated symptoms, like dysmenorrhea, dyspareunia, and infertility [3,4,5], significantly diminish the quality of life for affected individuals, causing both psychological and physical stress [6]. The diagnosis of AM is often delayed and therapy is confined to symptomatic approaches due to the uncertainty of the pathogenesis [7,8,9].

Metaplasia, translocation, and stem cell theory are the main hypotheses for the pathogenesis of AM. Due to the extraordinarily high regenerative potential of the endometrium, stem cell involvement of the basalis has been assumed [10,11,12]. The hypothesis that stem cells are located in the basal endometrium is supported by the knowledge that, during menstruation, only the functionalis ablates and post-ablation new endometrial tissue are able to develop [10,12,13]. These cells could migrate towards functionalis and be the origin of endometrial regeneration. It has been suggested that stem cells found in the basal endometrium may be the origin of AM [14,15]. This displacement may be due to tissue injury [14] or the increase in matrix metalloproteinases (MMP-2, MMP-9) promoting the migration of stem cells [16,17]. As these stem cells are thought to play a role in AM development, it is crucial to understand the factors and mechanisms that lead to their differentiation.

In 2015, Ibrahim et al. [18] introduced a new cell type discovered during their studies on the alteration of the endometrial–myometrial junctional zone (EMJZ)—the distinct, hormone-dependent endometrial–myometrial interface was believed to be a crucial part of disease development. The EMJZ is composed of the basal endometrium together with the inner one-third of the underlying myometrium [19]. This new cell type was characterized by a translucent cytoplasm, and they were thus named pale cells. Pale cells were predominantly found in the basal part of the endometrial glandular epithelium with partial/total cell border detachment and a lack of desmosomes [18]. Additionally, active protrusions (pseudopods) of individual cells into the underlying stroma were only observed in AM patients, indicating that pale cells were migrating and translocating into the underlying stroma. This unique behavior suggested a potential role in the development of AM [18].

This preliminary investigation suggested that pale cells were not classified as either leukocytes or macrophages (lacking the corresponding immunohistochemical markers) and are largely presumed to be stem cell-like. However, the specific cell type of pale cells remains unclear. In this study, after identifying pale cells, we examined the expression of Musashi-1 (MSI-1; an RNA-binding protein and a marker of neural stem cells and progenitor cells), SRY-box transcription factor 2 (SOX-2; a transcription factor and a marker of embryonic stem cells, neural stem cells, and pluripotent cells), stage-specific embryonic antigen-1 (SSEA-1; a glycoprotein—carbohydrate antigen and a marker of pluripotent stem cells and early germ cells), and E-cadherin (a cell adhesion molecule and a marker of epithelial cells and epithelial stem cells) in both AM and non-AM uterus tissues. The aim was to further characterize pale cells immunohistologically using different stem cell markers.

## 2. Materials and Methods

### 2.1. Patient and Sample Collection

Altogether, the samples of 60 enrolled patients were collected at the Department of Obstetrics and Gynecology, University Hospital Münster (2016–2017) and Charité (2012–2014). Of these patients, 34 underwent surgery following the initial diagnosis of AM based on ultrasonography, while 26 were initially diagnosed with other benign gynecological diseases such as myoma. All patients met surgical indications. Patients over the age of 50 or those with suspected lesions of malignancy were excluded. Endometrial samples were extracted immediately after hysterectomy from the upper one-third of the uterine cavity, specifically from the anterior/posterior wall, the primary site affected by AM and the most susceptible to cyclic tissue injury, according to TIAR theory. Specimens were taken so that both the endometrium and the inner layer of the myometrium were included in each specimen. In the end, 25 tissue samples were included in the AM group and 15 in the non-AM group (Table 1).

The study was approved by the ethics committee at the Institutional Review Board of Westfällsche Wilhelms University’s Medical Faculty (1 IX Greb) and Charité Medical University (EA4/036/12). All patients gave their consent prior to commencement.

### 2.2. Immunohistochemistry

Tissue samples were directly fixed with a 4% formaldehyde solution for a maximum of 72 h and, following dehydration and xylene addition, were embedded in paraffin. Sections of 3 µm thickness were mounted on glass slides and deparaffinized in xylene, followed by a graded ethanol series in descending concentrations. A citrate buffer with PBS (DAKO target retrieval solution 10× concentrate, Agilent Technologies, Santa Clara, CA, USA) and peroxidase block (peroxidase block from the DAKO EnVision TM + system, Agilent Technologies, Santa Clara, CA, USA) were applied. After blocking with 10% bovine serum albumin (BSA) for 30 min at room temperature, sections were incubated overnight with the primary monoclonal antibodies mouse anti-human MSI-1 (1:250, MAB2628, R&D Systems, Bio-Techne, Minneapolis, MN, USA), mouse anti-human SOX-2 (1:50, MAB2018R, R&D Systems, Bio-Techne, Minneapolis, MN, USA), mouse anti-human SSEA-1 (1:100, sc-101462, Santa Cruz Biotechnology, Dallas, TX, USA), and mouse anti-human E-cadherin (1:200, #610404, BD Bioscience, Franklin Lakes, NJ, USA) at 4 °C. Sections were stained with secondary antibodies (DAKO EnVision TM anti-mouse #K5007, Agilent Technologies, Santa Clara, CA, USA and VECTASTAIN ABC anti-mouse kit, VEC-PK-4002, BIOZOL, Eching, Germany) for 30 min. Then a suitable staining medium (DAKO AEC substrate chromogen from DAKO EnVision TM, Agilent Technologies, Santa Clara, CA, USA) was added to the section for an incubation of 5–10 min. After washing, counterstaining was performed with hematoxylin (Merck KG a.A., Darmstadt, Germany) for 10 s. Control sections without the specific primary antibody were processed as negative controls.

All sections were examined under a light microscope (Carl Zeiss Axiovert 100 microscope, Carl Zeiss, Oberkochen, Germany) with its corresponding camera (Carl Zeiss Axio Cam MRc, Carl Zeiss, Oberkochen, Germany) and software (AxioVision, version 4.8, Carl Zeiss, Oberkochen, Germany). In the EMJZ area, 10–15 fields were randomly selected at 32× magnification and analyzed by 2 investigators (V.H. and M.G.I.) independently using a modified immune reactive score (IRS) system previously described by Ibrahim et al. [18]. The staining intensity in both the endometrium (functionalis, basalis, and stroma) and the inner myometrium was scored as none = 0, weak = 1, moderate = 2, or strong = 3. The extent of staining was measured by an estimated percentage of immunopositive cells from 0 to 100. The mean value of the multiplied intensity and percentage determined the final IRS. In addition, depending on the number and size of the lesions, up to five randomly selected visual fields of foci were evaluated. Regarding expression levels, an IRS of 0–1 was classified as no expression (−), 1–100 as weak expression (+), 100–200 as moderate expression (++), and 200–300 as strong expression (+++). Pale cells were analyzed by number per field of view (high-power field—hpf).

### 2.3. Immunofluorescence

Sections for immunofluorescence were deparaffinized, antigen retrieved, protein blocked (as described for immunohistochemistry), and interspersed with 0.1% TritonX-100 during incubation. Primary monoclonal antibodies for mouse anti-human MSI-1 (1:250, MAB2628, R&D Systems, Bio-Techne, Minneapolis, MN, USA), mouse anti-human SOX-2 (1:50, MAB2018R, R&D Systems, Bio-Techne, Minneapolis, MN, USA), mouse anti-human SSEA-1 (1:100, sc-101462, Santa Cruz Biotechnology, Dallas, TX, USA), and rabbit anti-human E-cadherin (1:200, #3195, Cell Signaling Technology, Danvers, MA, USA) were applied overnight at 4 °C.

Sections were then incubated with the secondary antibodies Alexa Fluor 555 (rabbit anti-mouse IgG, 1:500, Thermo Fisher Scientific, Waltham, MA, USA) and Alexa Fluor 488 (donkey anti-rabbit IgG, 1:500, Thermo Fisher Scientific, Waltham, MA, USA) for 30 min followed by a 1-min incubation with 4’, 6-diamidine-2-phenylidol (DAPI, 1:10,000) before applying the mounting medium (DAKO Fluorescent Mounting Medium, Agilent Technologies, Santa Clara, CA, USA) and sealing with coverslips. Control sections not treated with the specific primary antibody were used as negative controls. The sections were examined using the fluorescence microscope Leica DMI3000 (Leica Microsystems, Wetzlar, Germany).

### 2.4. Immunoelectron Microscopy

Fixation, dehydration, and embedding of samples in a resin mixture were performed as described by Ibrahim et al. [18]. Using an ultramicrotome, ultrathin sections were cut, placed on copper grids, and stained with 2% uranyl acetate for 10 min. For immunogold electron microscopy, ultrathin sections were incubated with 100 mM glycine in PBS for 2 min, washed with PBS, and blocked with 2% (*w*/*v*) BSA in PBS. For ultrathin (80 nm) sectioning, embedding was performed in epoxy resins and was followed by heating for 45 s on an electric hot plate set to 80 °C. For immunolabeling, grids were incubated for 1 h at room temperature on drops of primary antibodies (anti-MSI-1 diluted to 1:100 and anti-SSEA-1 diluted to 1:50) in PBS containing 2% (*v*/*v*) BSA-c (Aurion, Wageningen, The Netherlands) and 0.025% (*v*/*v*) Tween 20. After washing with the same solution, ultrathin sections were incubated with secondary antibodies conjugated with gold particles. Immunogold labeling was performed with 18 nm gold-conjugated immunoglobulins. After washing with distilled water, ultrathin sections were negatively stained with 2% (*w*/*v*) uranyl acetate for 10 min. Electron micrographs were taken at 60 kV with a Phillips EM-410 electron microscope using imaging plates (Ditabis, Pforzheim, Germany).

### 2.5. Statistical Analysis

The results were evaluated using GraphPad Prism 9.5 software (GraphPad Software, Inc., Boston, MA, USA), and the data was analyzed with the Kruskal–Wallis test. Differences were considered significant if the *p*-value < 0.05.

## 3. Results

### 3.1. Immuno-Expression of E-Cadherin

**Figure 1:**
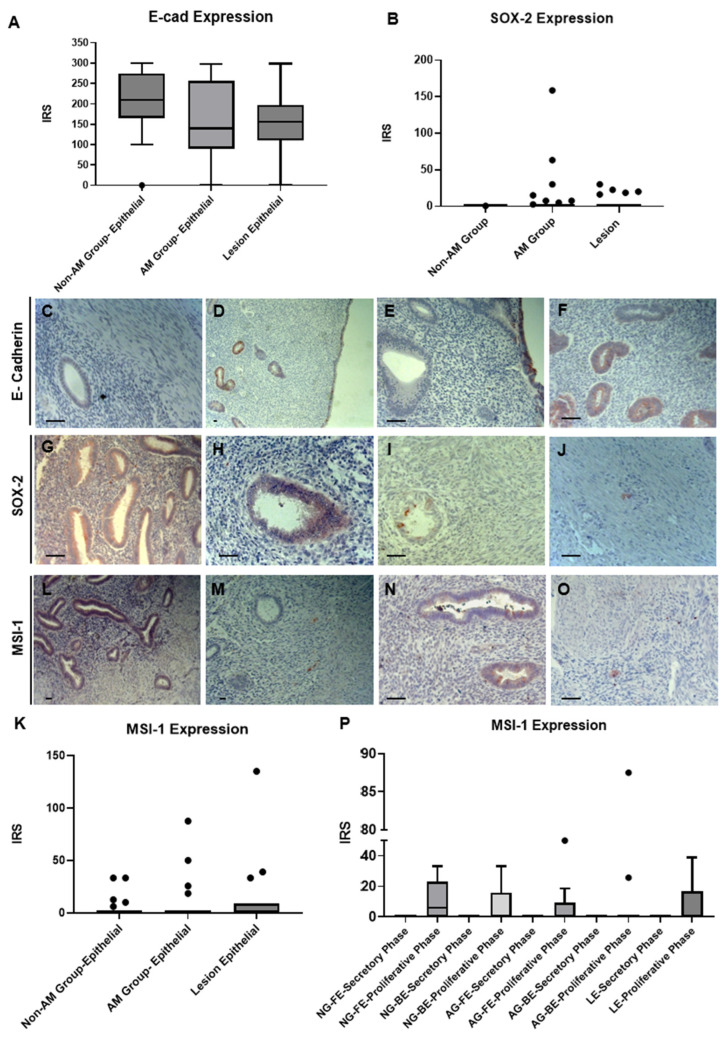
The immune expression pattern of E-cadherin, MSI-1, and SOX-2 in adenomyosis tissues. (**A**) E-cadherin expression in the epithelium of the non-AM group, AM group (eutopic), and in lesions (**B**) SOX-2 expression in the non-AM group, AM group, and lesions. (**C**,**D**) E-cadherin expression in both a lesion and the basalis region of the AM group within the same tissue section. (**E**,**F**) E-cadherin expression in the functionalis of the AM group. (**G**) SOX-2 expression in the basalis of the AM group. (**H**,**I**) SOX-2 expression in a lesion. (**J**) SOX-2 expression in the myometrium in the AM group. (**K**) MSI-1 expression in the epithelium of the non-AM group, AM group, and in lesions. (**L**,**N**) MSI-1 expression in the basalis of the AM group (**M**) MSI-1 expression in the stroma of lesions. (**O**) MSI-1 expression in the myometrium of the AM group. (**P**) MSI-1 expression in epithelium across different groups. NG = non-AM group, AG = AM group, LE = lesion epithelium, FE = functionalis epithelium, BE = basalis epithelium. Data were assessed using the Kruskal–Wallis test, and significant differences were considered at *p*-values of <0.05. Scale bar = 50 um.

E-cadherin immune expression was exclusively seen in the eutopic endometria of both AM and non-AM uteri and in the ectopic endometria of AM uteri but not in the stroma or myometria of either group. Though statistically insignificant (*p* = 0.055; Figure 1A), the non-AM group tended to have higher IRS values in the epithelia compared with the AM group. The immuno-expression of E-cadherin was also carried out with different cycles and areas in both groups (Supplementary Figure S1A,B). Notably, in the AM group, some glands exhibited either weak or completely absent E-cadherin expression, indicating a significant reduction or loss of this protein. This distinct pattern of diminished or absent E-cadherin expression was observed in only 2 out of 21 samples of eutopic endometria. The presence of E-cadherin-positive and -negative glands in the same sample (same slide) was observed in 10 out of the 25 examined AM ectopic tissues (Figure 1C–F). As pale cells lack desmosomes, they are expected to present as E-cadherin-negative epithelial cells residing in the glandular tissue (eutopic and ectopic).

### 3.2. Immuno-Expression of SOX-2

In the AM group, 32% of tissue samples contained SOX-2-positive cells, while these were observed in only 8% of the non-AM samples. However, the overall expression of SOX-2 was weak in both groups (Figure 1B), with no positive expression seen in the stroma or myometrium in the non-AM group. In contrast, in the AM group, there were positive SOX-2 cells present in these areas, although they were present in very small numbers (Figure 1G–J).

### 3.3. Immuno-Expression of MSI-1

MSI-1, a stem cell marker known to be dysregulated in endometriosis and endometrial cancer [20], was expressed in around two-thirds of the tissue samples in the AM group, in contrast to only about one-third in the non-AM group. There was no ascending trend observed in the AM group (*p* = 0.333), as shown in Figure 1K. The majority of MSI-1-positive cells were located in the endometrial epithelium, with fewer positive cells observed in the stroma and myometrium (Figure 1L–O). MSI-1 exhibited a higher expression tendency in proliferative epithelial cells compared to the secretory phase, although this difference was not significant (*p* = 0.400; Figure 1P).

### 3.4. SSEA-1 Is Upregulated in AM Lesions

**Figure 2:**
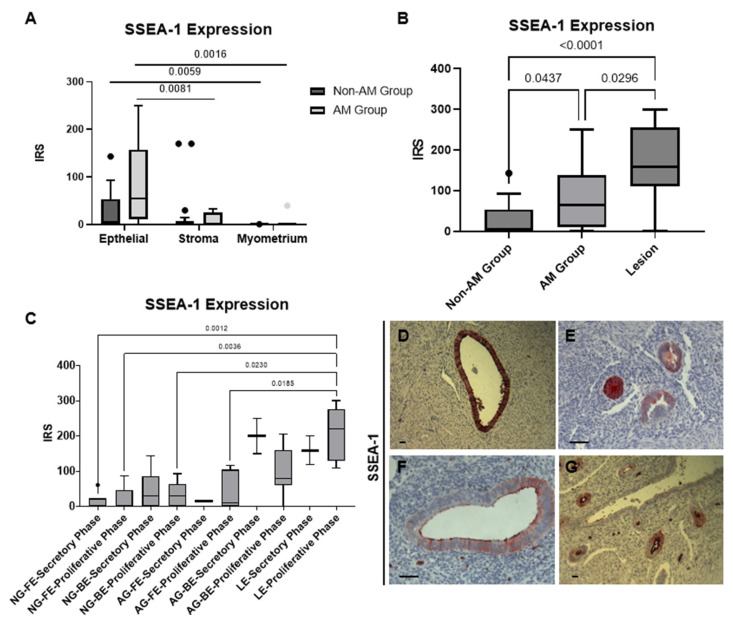
SSEA-1 is upregulated in adenomyosis lesions. (**A**) SSEA-1 expression in the non-AM and AM groups. (**B**) SSEA-1 expression in the epithelium of the non-AM group, AM group (eutopic), and in lesions. (**C**) SSEA-1 expression according to cycle phase and groups. (**D**–**F**) SSEA-1 expression in AM group lesions. (**G**) SSEA-1 expression in the functionalis of the AM group. NG = non-AM group, AG = AM group, LE = lesion epithelium, FE = functionalis epithelium, BE = basalis epithelium. Data were analyzed by the Kruskal–Wallis test. Significant differences were considered at *p*-value < 0.05. Scale bar = 50 um.

All tissue samples from the AM and non-AM groups were positive for the marker SSEA-1. The samples exhibited significantly stronger expression of SSEA-1 compared to the two markers MSI-1 and SOX-2. Positive cells were predominantly observed in the endometrial epithelium. The glandular epithelium in the AM group had significantly higher expression compared with the stroma (*p* = 0.0081) and myometrium (*p* = 0.0016; Figure 2A). No difference in expression was observed in the epithelium of the functionalis and basalis (*p* = 0.3677; Supplementary Figure S2). The AM group showed higher expression in lesions compared with the non-AM group (*p* < 0.0001) and eutopic epithelium (*p* = 0.0296). Similar SSEA-1 expression was also observed between the AM and non-AM groups (*p* = 0.0437; Figure 2B). Regarding different cycle phases, when compared with the lesion epithelium, an increase in SSEA-1 expression was only observed in the proliferative phase (Figure 2C). In the SSEA-1 staining, diffuse cytoplasmic expression of SSEA-1 was observed in the glandular epithelium of the lesions, as well as in the endometrium (Figure 2D–G).

The immune expression of different markers is summarized in Table 2.

### 3.5. Phenotypic Characterization of Pale Cells by Light Microscopy

**Figure 3:**
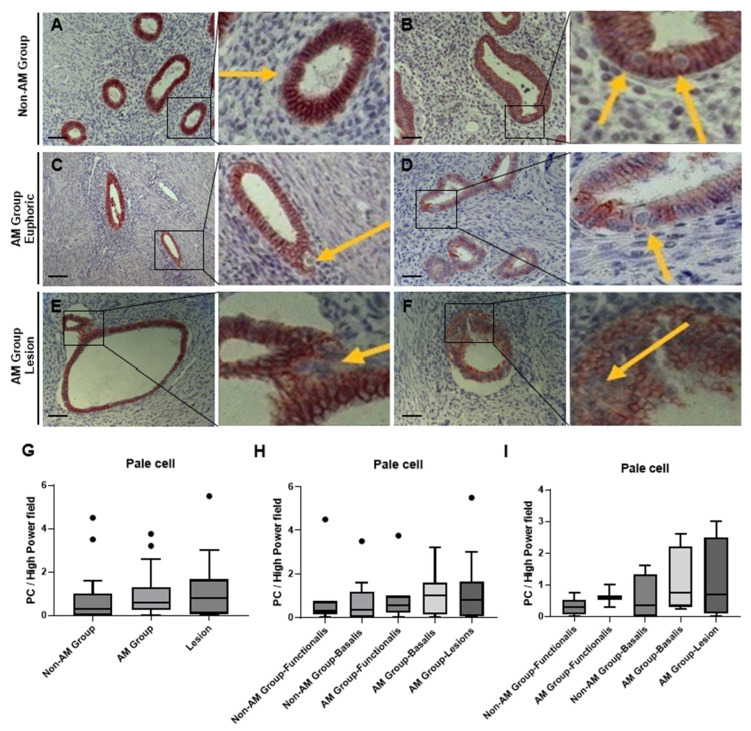
Pale cell recognition by light microscopy. (**A**,**B**) PCs in eccentric, central, and luminal localizations in the non-AM group. (**C**,**D**) Eccentrically and centrally arranged PCs in the AM group. The bright cytoplasm, large roundish morphology, and enlarged cell nucleus are recognizable. (**E**,**F**) PCs centered in endometrial and adenomyotic lesions of the AM group. (**G**) Pale cell number per field in the non-AM group, AM group (eutopic), and lesions. (**H**) Pale cell number per field in different layers in each group. (**I**) Pale cell number per field in the secretion cycle among different layers in each group. Data were analyzed by the Kruskal–Wallis test. Significant differences were considered at *p*-values < 0.05. Scale bar = 50 um.

Pale cells were observed under a light microscope in both the non-AM and the AM samples. The non-AM group contained approximately 20% less pale cell-positive tissue samples, despite the difference in the total number of pale cells not being significantly different (*p* = 0.46; Figure 3G). Pale cells were found to be localized within the glandular epithelium in both functionalis and basalis layers as well as in the lesions (Figure 3A–F).

Pale cells have a distinctive morphology: they appear as large, rounded cells with a markedly enlarged nucleus and exceptionally bright cytoplasm. These cells are predominantly solitary and only occasionally clustered. Within the glandular epithelium, pale cells reside either eccentrically (close to the basal membrane), centrally, or luminally (close to the luminal cavity). In most cases, pale cells were centered within the glandular epithelium. A luminal position was only seen in about 10% of the samples (Table 3).

The basalis shows an ascending trend (not significant) compared to the functionalis within each group (*p* = 0.7170, Figure 3H), particularly during the secretory phase (*p* = 0.5645, Figure 3I).

### 3.6. Immuno-Labeling of Ultrastructurally Identified Pale Cells Using Transmission Electron Microscopy

**Figure 4:**
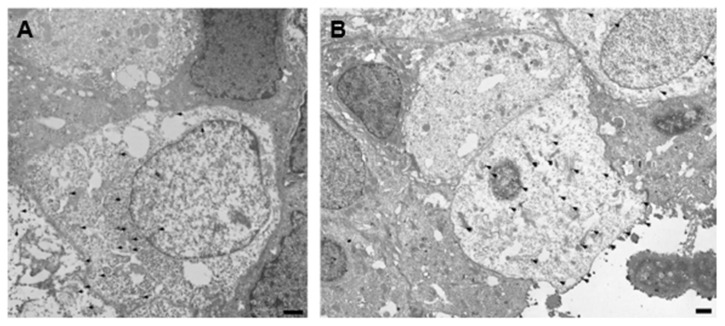
Immunoelectron microscopy revealed the expression of MSI-1 and SSEA-1 in pale cells. (**A**) Immunostaining for MSI-1 in the cytoplasm of electron-lucent cells. (**B**) Immunostaining for SSEA-1. Electron-dense cytoplasmic structures, as well as the cell surface, were labeled. Representative gold particles are highlighted by arrow heads. Scale bar = 500 nm.

At the ultrastructural level, pale cells were originally characterized by the presence of heterochromatic nuclei, mitochondrial and ribosomal abundance in the cytoplasm, and a more electro-lucent cytoplasm that distinguishes these cells from surrounding glandular epithelial cells [18]. To explore the expression of the stem cell markers SSEA-1 and MSI-1 in pale cells, we performed immunoelectron microscopy to complement our light microscopy investigations. The expression of MSI-1 was observed in the cytoplasm of electron-lucent cells that were likely pale cells (Figure 4A). Additionally, SSEA-1 expression was observed in the cytoplasm and on the cell surface of pale cells. Strikingly, SSEA-1 antibody-labeled electron-dense tube-like structures were seen within the electron-lucent cytoplasm, as well as labeled vesicle-like structures close to the cell surface (Figure 4B). Overall, these data confirm the expression of the stem cell-associated markers MSI-1 and SSEA-1 in pale cells as defined by their previously described ultrastructure [18].

### 3.7. Colocalization of E-Cadherin Negative and SOX-2/MSI-1/SSEA-1 Positive Cells

**Figure 5:**
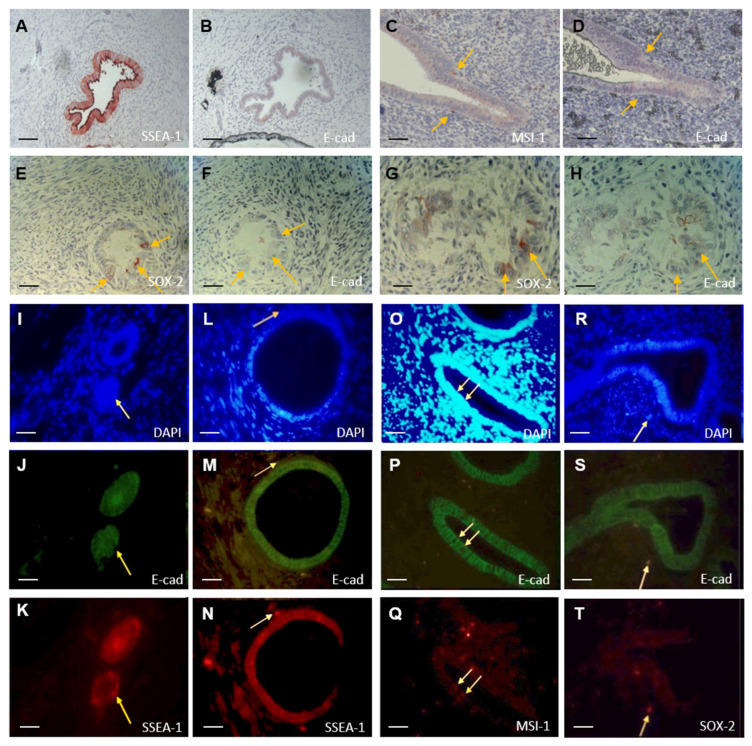
Colocalization of E-cadherin-negative and SOX-2-, MSI-1-, or SSEA-1-positive cells. (**A**) SSEA-1 immunohistochemistry staining in the lesion. (**B**) E-cadherin immunohistochemistry staining in the same lesion. (**C**) MSI-1 immunohistochemistry staining in the lesion. (**D**) E-cadherin immunohistochemistry staining in the same lesion. (**E**,**G**) SOX-2 immunohistochemistry staining in the lesion. (**F**,**H**) E-cadherin immunohistochemistry staining in the same lesion. (**I**–**K**) Immunofluorescence staining of the basal epithelium in the AM group. (**L**–**N**) Immunofluorescence staining of a lesion in a stroma in the AM group. (**O**–**Q**) Immunofluorescence staining of the functionalis in the AM group. (**R**–**T**) Immunofluorescence staining of stromal basalis in the AM group. Scale bar = 50 um.

All sections of the AM samples were examined if they showed cells lacking expression of E-cadherin (pale cell expression patterns) that were also positive for the aforementioned stem cell markers. Isolated colocalization of SSEA-1-positive and E-cadherin-negative cells were detected within lesions using immunohistochemistry (Figure 5A,B). In some sections, colocalization was observed in individual cells. However, completely E-cadherin-negative glands were predominantly seen in the lesions, which also exhibited strong SSEA-1 expression.

Clear colocalization was rarely detected in MSI-1 staining. An isolated MSI-1-positive cell was observed within a lesion′s marginal area of the glandular epithelium. This cell showed slightly diffuse MSI-1 expression and no E-cadherin expression (Figure 5C,D).

Increased colocalization was especially evident in the lesions of the AM group with SOX-2 and E-cadherin staining. Isolated SOX-2-positive cells were present (Figure 5E,F) and, in the corresponding sections of other tissue samples, these cells showed no E-cadherin expression.

After the immunohistochemistry staining provided an initial indication of possible colocalization, double immunofluorescence was employed. Cells that were negative for E-cadherin and positive for SSEA-1 were observed in the glands of the basalis. In one patient, these cells appeared to protrude from the epithelial lining of the glands into the basalis (Figure 5I–K). Colocalization was also observed in the basal stroma (Supplementary Figure S3A).

In addition, colocalization of SSEA-1-positive and E-cadherin-negative cells was also seen in the stroma of the functionalis and the lesions (Figure 5L–N, Supplementary Figure S3B). The staining also revealed a high occurrence of SSEA-1-positive cells in the luminal and apical regions of the glands, as was previously seen in immunohistochemistry staining (Supplementary Figure S3C). In the functionalis and basalis, E-cadherin-negative and MSI-1-positive cell colocalization was found (Figure 5O–Q). This colocalization was observed in both the epithelium and the stroma (Supplementary Figure S4A). No colocalization was found within the lesions in the examined sections.

When examining the colocalization of SOX-2-positive and E-cadherin-negative cells, we found an overlapping presence within the functionalis and basalis, but only in the stroma (Figure 5R–T). No positive cells were observed within the epithelium in the two sections examined (Supplementary Figure S4B).

## 4. Discussion

As far as we know, this is the first instance of visualizing pale cells using immunohistochemistry and immunoelectron microscopy following their identification by Ibrahim et al. in 2015 [18]. Pale cells, characterized by their bright appearance and larger, rounded nucleus shape, were observed in all patients with AM and in 80% of tissue samples in the non-AM group. Given the consistency in location and the “single cell” feature, it is reasonable to speculate that the cells we observed under the microscope were pale cells. Notably, immunoelectron microscopy demonstrated the expression of both SSEA-1 and MSI-1 in pale cells, confirming our findings at the ultrastructural level.

Pale cells were characterized by detachment from the basement membrane and the absence of desmosomes, with motility verified primarily through the absence of E-cadherin expression. While a slight decrease in E-cadherin expression was noted in the AM group, no significant difference was observed between the lesion and eutopic epithelium. However, E-cadherin-negative glands were notably present in the AM group, particularly within lesions. This finding is consistent with previous studies [21,22,23,24], like that of Bartley and Poncelet, who suggested that variations in E-cadherin expression may denote different stages of lesion development. Despite this, the presence of E-cadherin-negative/pale cells in the epithelium and lesions supports the hypothesis that these cells are freely motile, can invade other compartments, and may contribute to the development of AM lesions.

Furthermore, the higher presence of pale cells in the basal epithelium rather than in the functional epithelium of the AM group, when compared to the non-AM group, supports the results found by Ibrahim et al. [18]. Moreover, the absence of variations among the cycle phases indicates that the epithelial-to-mesenchymal transition (EMT) in this scenario is not influenced by elevated estrogen levels. Instead, it is plausible that the EMT is triggered by signals induced by uterine injuries [14]. As proposed by Ibrahim et al. [18], these injuries could manifest as “ultramicro ruptures”, facilitating the migration of E-cadherin-negative/pale cells into the stroma and then further into the myometrium.

In our study, SSEA-1 staining revealed expression of this stem cell marker in nearly the entirety of the epithelium across all tissue samples. Valentjin et al. [25] used the SSEA-1 surface marker to identify the basal endometrium as the hypothesized origin of the endometrial stem cells. Their findings showed that SSEA-1 expression was mainly present in the epithelial compartment across all groups, with no detectable expression in the stromal tissue. Our investigation uncovered a significant difference between the basal and functional epithelium within the AM group. This marks the first instance of such a disparity, suggesting a potential migration towards the myometrium. The colocalization of SSEA-1-positive/E-cadherin-negative cells further bolsters this hypothesis.

Furthermore, Tempest et al. [26] reported SSEA-1-positive cells in the luminal epithelium, with weaker expression in the functionalis and stronger expression in the basalis, independent of cycle-related hormonal fluctuations. They proposed the existence of two distinct stem cell pools within the human endometrium: one in the basal epithelium for endometrial regeneration and another in the luminal epithelium to facilitate implantation during embryogenesis. Our findings align with this observation, as we also noted luminal expression of SSEA-1, supporting the presence of distinct stem cell populations.

The presence of stem cells in the basal glandular epithelium of the endometrium was initially described by Gargett et al. [10] as a characteristic stem cell position. They suggested that undifferentiated cells within the basal glandular epithelium, particularly in the functionalis, may serve as the origin for endometrial epithelium regeneration during the menstrual cycle. This theory is further supported by Nguyen and Gargett [27], who detected N-cadherin- and SSEA-1-positive cells in the EMJZ. While these markers were present in the basalis, they were notably absent in the functionalis, indicating a hierarchical organization from the EMJZ to the functionalis.

All data regarding MSI-1 in the present study demonstrate no statistical significance, suggesting that they should be interpreted solely as a trend and are not sufficient to reliably establish that MSI-1-positive cells correspond to those capable of migrating into the myometrium. Double immunostaining, unfortunately, seldom revealed MSI-1-positive cells colocalizing with E-cadherin-negative cells. Such cells were only sporadically observed in the stroma of the basalis in both tissue samples, with colocalization within the epithelium being observed in a single visual field within the functionalis. It is important to note that MSI-1-positive cells showed an increased concentration of ß-catenin, indicating potential colocalization with E-cadherin-negative cells. This suggests a significant influence of ß-catenin on the epithelial–mesenchymal transition or reduced E-cadherin expression [28,29].

The heightened occurrence of MSI-1-positive cells observed in AM patients, both in our study and in the literature [20,30], implies an involvement of MSI-1 cells in AM pathogenesis. Furthermore, the presence of MSI-1 positive (single) cells in the stromal and myometrial regions of the AM group and in lesions [20,30,31] suggests that these cells could actively migrate individually, potentially reaching the myometrium via cell migration and serving as the origin of adenomyotic lesions.

Götte et al. demonstrated colocalization of MSI-1-positive cells with telomerase and Notch-1 expression using double immunofluorescence [20]. Another study showed a correlation between Ki-67 and MSI-1-positive cells in sheep mammary tissue, indicating stem cell characteristics in these cells [32]. The presence of positive MSI-1 cells in the basal eutopic endometrial epithelium, as observed in this study, further supports the assumption of stem cell characteristics. While this finding was not statistically significant compared to the functionalis, in our case, results from the literature suggest a significant difference [20].

No statistical significance was observed for SOX-2 in the present studies, indicating that the following results should be interpreted only as trends. Hapangama et al. reported an inability to detect positive SOX-2 expression in the human endometrium and in endometriotic lesions [33]. Conversely, Götte et al. utilized qPCR to identify positive SOX-2 mRNA in all the tissue samples they examined, including six with adenomyotic foci [34]. In our findings, epithelial expression of SOX-2 tended to be localized in the basal regions of the epithelium. However, SOX-2-positive cells colocalizing with E-cadherin-negative cells were only observed in the stroma of the basalis and functionalis of the endometrium in the AM group. This observation may suggest a role for SOX-2 in AM pathogenesis.

In the present study, occasionally unexpected or significantly deviating values were encountered, possibly attributed to the relatively small patient pool available for analysis. This limitation partly stems from the infrequent occurrence of hysterectomies. Consequently, access to only a limited number of full-thickness uterine sections, especially during the secretory phase, was common. Additionally, providing four tissue samples per patient in both AM and non-AM groups was not always feasible. Future studies involving a larger patient population would be beneficial to validate the assumptions made in this study. Investigating the colocalization of stem cell markers with epithelial-mesenchymal transition markers such as Snail, Twist, or ZEB1, as well as exploring the expression of markers like Ki-67 or p63 in E-cadherin-negative cells, could provide insights into proliferative activity. Furthermore, examining SSEA-1-expressing cells could involve additional parameters, considering patient data, types of adenomyosis, or clinical symptoms. It would also be essential to ascertain whether SSEA-1-positive cells are leukocytes or stem cells from the bone marrow.

Examining Oct-4 and Nanog expression in adenomyotic tissue samples could offer a comprehensive profile of potential stem cell properties. Given the inconsistent literature on the marker MSI-1, further investigations in this area are warranted. Additionally, given the clear colocalization of SOX-2 and E-cadherin-negative cells, exploring SOX-2 in a larger patient pool could yield valuable insights. In conclusion, the specific morphology of pale cells allowed for their initial visualization via immunohistochemistry and microscopy. The presence of pale cells in all AM group tissue sections and increased SSEA-1-positive cells and reduced E-cadherin expression in the basalis indicate the involvement of epithelial–mesenchymal transition/pale cells in AM. SSEA-1 positive cells colocalized with E-cadherin-negative cells suggest that these cells possess stem cell characteristics. Immunofluorescence showed a detached SSEA-1+/E-cadherin- cell, supporting the hypothesis that migrating pale cells contribute to AM. The significant increase in SSEA-1-positive cells in the AM group’s basal epithelia suggests active migration and potential stem cell properties in these cells. However, due to a lack of statistical significance and inconsistent results, MSI-1 and SOX-2 markers did not provide a clear indication that these cells are the cause of AM.

## Figures and Tables

**Table 1:** Clinical data of the patients included in this study.

Group	Age	Diagnosis	Cycle	GPA	Symptoms
AM Group	44	Adenomyosis uteri	p	000	dysmenorrhea
	47	Adenomyosis uteri	p	NI	-
	42	Adenomyosis uteri	p	102	defecation problems
	43	Endometriosis	p	321	lower abdominal pain
	36	Adenomyosis uteri	p	422	-
	43	Adenomyosis uteri	p	413	dysmenorrhea, hypermenorrhea
	47	Endometriosis	*	220	dysmenorrhea, hypermenorrhea
	50	Adenomyosis uteri	s	321	hypermenorrhea
	37	Adenomyosis uteri	p	000	dysmenorrhea
	47	Adenomyosis uteri	p	110	hypermenorrhea
	30	Adenomyosis uteri	s	000	dysmenorrhea, hypermenorrhea
	37	Adenomyosis uteri	p	000	hypermenorrhea, lower abdominal pain
	41	Adenomyosis uteri	s	954	dysmenorrhea, hypermenorrhea
	34	Adenomyosis uteri	a	000	-
	37	Adenomyosis uteri	p	NI	-
	44	Adenomyosis uteri	s	000	hypermenorrhea
	36	Adenomyosis uteri	p	413	dysmenorrhea, hypermenorrhea
	41	Adenomyosis uteri	p	220	dyspareunia, hypermenorrhea
	41	Adenomyosis uteri	p	000	-
	41	Adenomyosis uteri	p	220	dysmenorrhea
	30	Adenomyosis uteri	p	NI	dysmenorrhea
	39	Adenomyosis uteri and endometriosis	*	000	-
	47	Endometriosis	a	110	dysmenorrhea, dyspareunia, hypermenorrhea
	45	Adenomyosis uteri and endometriosis	*	000	dysmenorrhea, lower abdominal pain
	45	Endometriosis	*	000	dysmenorrhea, dyspareunia
Non-AM Group	46	Uterus myomatous	s	120	lower abdominal pain
	47	Uterus myomatous	p	NI	postmenopausal syndrome
	37	Uterus myomatous	s	211	-
	45	Vascular anomaly	s	211	dysmenorrhea
	48	Uterus myomatous	p	000	hypermenorrhea
	50	Uterus myomatous	p	NI	-
	43	Uterus myomatous	s	000	dyschezia
	50	Uterus myomatous	*	220	micturation disorders
	32	Cervical intraepithelial neoplasia III	p	541	-
	44	Uterus myomatous	p	330	hypermenorrhea
	17	Uterus didelphys	p	000	-
	48	Uterus myomatous	p	220	lower abdominal pain, hypermenorrhea
	41	Chronic endocervicitis	s	220	-
	50	Uterus myomatous	s	NI	-
	44	Uterus myomatous	s	NI	-

Cycle phase: a = atrophic, s = secretory, and p = proliferative. GPA: G (gravidity) = pregnancy, P (parity) = birth, and A (abortion) = miscarriage. * = cycle phase could not be determined histologically. NI = no information.

**Table 2:** Immune expression of different markers in various tissues in both AM and non-AM groups.

Marker	Eutopic Epithelial Cells	Ectopic Epithelial Cells	Stromal Cells	Myometrial Cells
E-cadherin Non-AM	+++	#	−	−
E-cadherin AM	++	++	−	−
SOX-2 Non-AM	−	#	−	−
SOX-2 AM	−	−	−	−
MSI-1Non-AM	−	#	−	−
MSI-1 AM	−	−	−	−
SSEA-1 Non-AM	+	#	+	−
SSEA-1 AM	+	++	−	−

Strong expression (+++), moderate expression (++), weak expression (+), no expression (−), not related (#).

**Table 3:** Location and total occurrence of pale cells by tissue section.

	Lesion	Basalis	Functionalis
Centered	9/22	11/18	9/13
Eccentric	8/22	4/18	3/13
Luminal	5/22	3/18	1/13
Total Occurrence	22/25	18/21	13/14

## Data Availability

All data used and analyzed in this study are available from the corresponding author upon reasonable request.

## Supplementary Materials

The following supporting information can be downloaded at: https://www.mdpi.com/article/10.3390/biom14111355/s1, Figure S1: E-cadherin expression according to cycles and groups; Figure S2: SSEA-1 expression in tissue sections; Figure S3: SSEA-1 expression in the AM group; Figure S4: MSI-1 expression in the AM group.
