# Supplementary material for: Characterization of E-Cadherin, SSEA-1, MSI-1, and SOX-2 Expression and Their Association with Pale Cells in Adenomyosis
Source: Biomolecules. 2024 Oct 24;14(11):1355. doi: 10.3390/biom14111355 (PMC11591647; doi:10.3390/biom14111355)
Supplement: Supplementary file 1 [file biomolecules-14-01355-s001.zip › biomolecules-3252384-supplementary.pdf]

Supplementary Figures

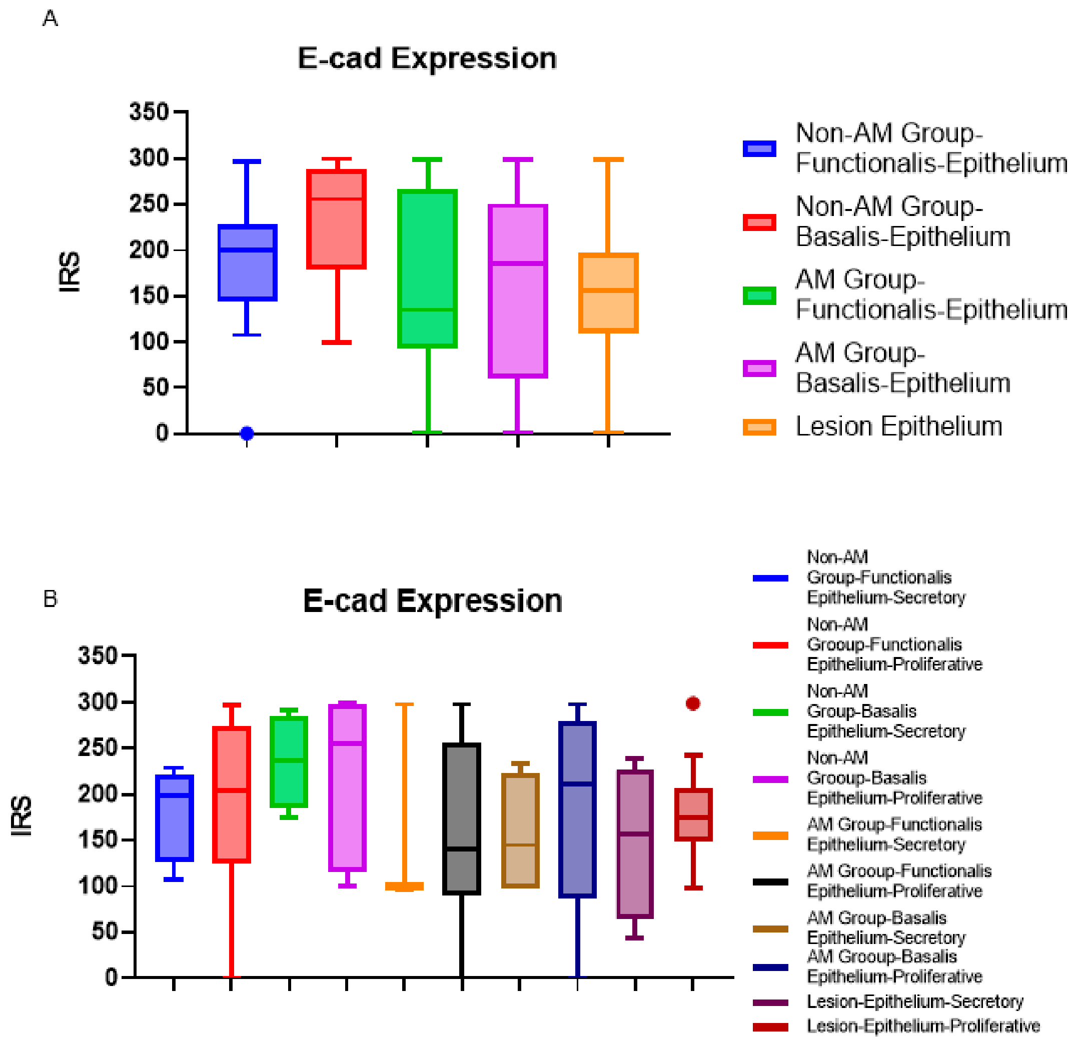

Figure S1. E-cad expression according to cycles and groups. Data was analyzed by the Kruskal-Wallis test. Significant differences were considered for P- value <0.05.

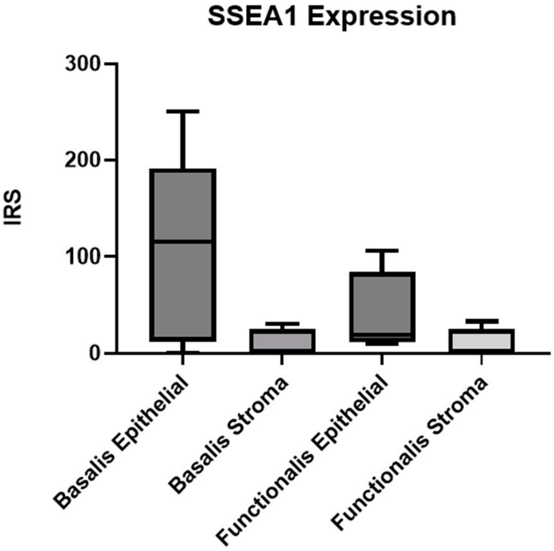

Figure S2. SSEA1 expression in tissue sections. Data was analyzed by the Kruskal-Wallis test. Significant differences were considered for P- value <0.05.

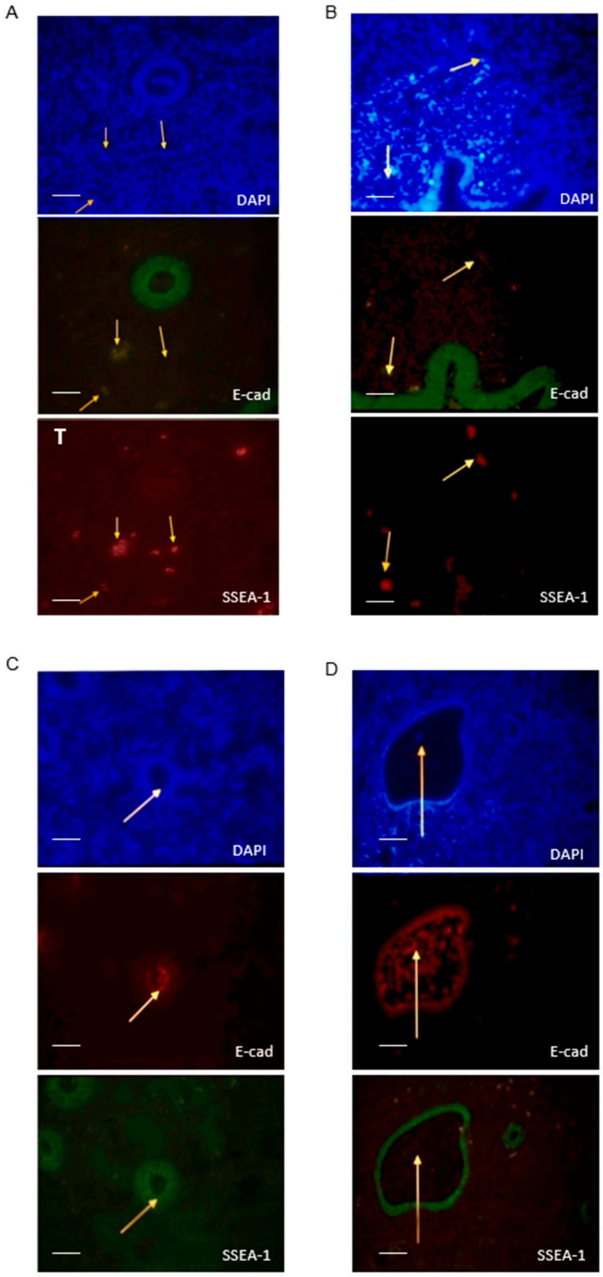

Figure S3. SSEA-1 expression in AM group. (**A**) Immunofluorescence staining of the basalis epithelium in AM group. (**B**) Immunofluorescence staining of the functionalis stroma in AM group. (**C**) Immunofluorescence staining of the basalis epithelium in luminal staining of the glandular secretion in AM group. (**D**) Immunofluorescence staining of the basalis epithelium in apical structure in AM group. Scale bar = 50um.

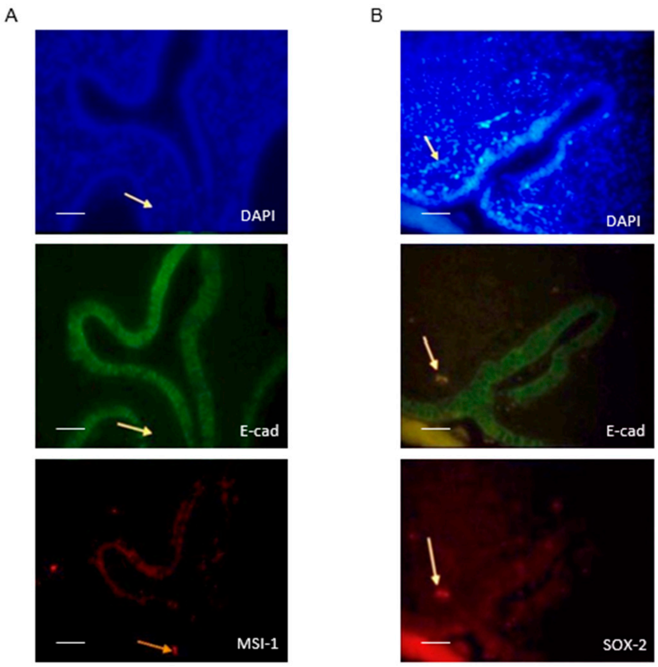

Figure S4. MSI-1 expression in AM group. (**A**) Immunofluorescence staining of the basalis stroma in AM group. (**B**) Immunofluorescence staining of the functionalis stroma in AM group. Scale bar = 50um.
